# S- and P-type cobra venom cardiotoxins differ in their action on isolated rat heart

**DOI:** 10.1590/1678-9199-JVATITD-2021-0110

**Published:** 2022-04-04

**Authors:** Alexey S. Averin, Mikhail V. Goltyaev, Tatyana V. Andreeva, Vladislav G. Starkov, Victor I. Tsetlin, Yuri N. Utkin

**Affiliations:** 1 Institute of Cell Biophysics, Federal Research Center Pushchino Scientific Center for Biological Research of the Russian Academy of Sciences, Pushchino, Russia.; 2 Shemyakin-Ovchinnikov Institute of Bioorganic Chemistry, Russian Academy of Sciences, Moscow, Russia.

**Keywords:** Cardiotoxin, Cobra venom, Contraction, Contracture, Myocardium, Perfused rat heart, Ventricular pressure

## Abstract

**Background:**

The cardiovascular system is one of the first systems to be affected by snake toxins; but not many toxins exert a direct effect on the heart. Cobra venom cardiotoxins are among those few toxins that attack the heart. Although the two cardiotoxin types (S and P) differ in their central-loop structure, it is not known whether they differ in their effect on the mammalian heart. We compared the effects of S- and P-type cardiotoxins, CTХ-1 and CTХ-2, respectively, from the cobra *Naja oxiana*, on the isolated rat heart*.*

**Methods:**

An isolated rat heart perfused according to the Langendorff technique was used in this study to investigate the activity of cardiotoxins CTX-1 and CTX-2. The following parameters were registered: the left ventricular developed pressure, calculated as the difference between systolic and diastolic pressure in the left ventricle, the end-diastolic pressure, the heart rate, time to maximal end-diastolic pressure (heart contracture), and time to depression of the heart contraction.

**Results:**

Both cardiotoxins at the concentration of 5 μg/mL initially produce a slight increase in systolic intraventricular pressure, followed by its rapid decrease with a simultaneous increase in diastolic intraventricular pressure until reaching contracture. CTX-2 blocks cardiac contractions faster than CTX-1; in its presence the maximum diastolic pressure is reached faster and the magnitude of the developed contracture is higher.

**Conclusion:**

The P-type cardiotoxin CTX-2 more strongly impairs rat heart functional activity than the S-type cardiotoxin CTX-1, as expressed in its faster blockage of cardiac contractions as well as in more rapid development and greater magnitude of contracture in its presence.

## Background

Among the vast family of three-finger snake venom toxins, there is a group of proteins called cardiotoxins (CaTXs) or cytotoxins that exert damaging effects on the heart. In particular, CaTXs cause systolic heart arrest and induce a membrane leakage of cardiomyocytes. They are β-structured proteins consisting of 59 to 61 amino-acid residues with the structure stabilized by four disulfide bonds. This group of toxins is characterized by direct interaction with the membrane, leading to membrane depolarization and cell death (see reviews [1, 2]). 

**Figure 1.**
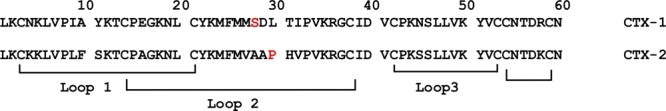
Amino-acid sequences of CTX-1 (UniProtKB accession # P01451 (3SA1_NAJOX)) and CTX-2 (UniProtKB accession # P01441 (3SA2_NAJOX)). Serine 28 (S) and proline 30 (P) residues are shown in red.

CaTXs are classified into two types, P and S: P-type includes CaTXs with a proline residue at position 30 of the amino-acid sequences and alanine at position 28 in most sequences, while S-type toxins have a serine residue at position 28 (Figure 1) and never contain proline at position 30, which is usually occupied by leucine, lysine or serine residues. The data available to date indicate that toxins of both types destabilize the lipid bilayer of anionic phospholipid-containing membranes, but with different efficiencies [3]; P-type toxins damage the lipid bilayer more severely. 

The effects of various CaTXs on myocardial tissue develop quite typically: the initial increase in contractility is followed by its suppression and by an increase in diastolic tension [4, 5]. At the same time, there are CaTXs that, at fairly high concentrations of tens of μM, produce an increase in the contraction force without the contracture development [4]. It should be noted that CaTXs affect the blood vessels as well, and this is done in two phases: the initial phase of relaxation, dependent on the endothelium, is then replaced by a slowly developing tonic contraction [6, 7]. However, there are practically no data on the similarities or differences in the effect of different types of CaTXs on the heart and blood vessels. 

Cytotoxins (cardiotoxins) 1 (CTX-1) and 2 (CTX-2) from the venom of the Central Asian cobra *Naja oxiana* belong to the S- and P-types, respectively [8]. In our recent work [9], we studied the effect of CTX-1 and CTX-2 on papillary muscle from the right ventricle of the rat heart. CTX-2 was found to show greater activity than CTX-1 [9]. However, in view of the tissue-specificity of the cardiotoxin effects [7, 10-12], it remained unclear whether these differences would extend to cardiotoxin action on such a complex object as a whole heart containing several types of tissues.

It was shown previously that CTX-2 at the concentration of 10 μg/mL produced a decrease in the amplitude of heart contractions, bradycardia and cardiac arrest in systole of an isolated frog heart, while CTX-1 caused arrhythmia, and at significantly higher concentrations also caused systolic cardiac arrest [8]. The explanation for these differences has not been clarified. Furthermore, the question of the applicability of the data obtained on the amphibian heart to mammalian physiology and, in particular, the human heart, remains unresolved. It should be noted that the effect of *Naja naja atra* cobra venom and some of its fractions, identified by the authors as cardiotoxic to the rat heart, was studied previously [5]. However, data on the composition of the investigated venom fractions were not given. In this work, we investigated the effects of individual well-characterized cardiotoxins, namely CTX-1 (S-type) and CTX-2 (P-type), on the contractile parameters of a whole rat heart perfused according to the Langendorff technique. 

## Methods

Cardiotoxins were purified from *Naja oxiana* cobra venom as described previously [3, 13], and their purity and structure were confirmed by HPLC and mass-spectrometry. 

Adult male Wistar rats (body weight, 200-250 g) were used for the experiments. The study was conducted according to the guidelines of the Declaration of Helsinki, Directive 2010/63/EU of the European Parliament and of the Council on the protection of animals used for scientific purposes (22 September 2010) and approved by the Biological Safety and Ethics Committee of the Institute of Cellular Biophysics (Instruction for the use of laboratory animals in the Institute of Cellular Biophysics №57 of 30.12.2011). Hearts were perfused using the Langendorff technique essentially as previously described [14]. Rats were anesthetized with sodium pentobarbital (50 mg/kg), the hearts were removed from the opened chest, immediately attached by the aorta to a cannula, and the retrograde perfusion of the isolated heart was performed under stable perfusion pressure of 75-80 mmHg with non-recirculating Tyrode solution containing (in mM): NaCl, 135; KCl, 4; MgCl_2_,1; CaCl_2_, 1.8; NaHCO_3_, 14.5; NaH_2_PO_4_, 1.8; glucose, 11; pH 7.4. The perfusion solution was aerated with 95% O_2_/5% CO_2_, while its temperature was held constant at 37 ± 0.1°C. Left ventricular pressure (LVP) was measured isovolumetrically using a latex balloon introduced into the left ventricle through an incision in the left atrium and inflated to a baseline diastolic pressure of 10 mmHg. The balloon was connected to the pressure transducer, and the diastolic, systolic, and pulse LVP were recorded and processed using the software PhysExp (Cardioprotect Ltd., Saint Petersburg, Russian Federation). 

After 30 minutes of equilibration, CTX-1 or CTX-2 was added to the solution and was maintained during the whole experiment at the concentration of 5 µg/mL. A group of 6 rats was used for CTX-1 measurements and another of 5 rats for CTX-2. The following parameters were registered: the left ventricular developed pressure (LVDP), calculated as the difference between systolic and diastolic pressure in the left ventricle; the end-diastolic pressure (LVEDP); the heart rate; time to maximum LVEDP (contracture of the heart) and time to depression of heart contractions (LVDP lower than 5 mmHg). In every experiment, the preparation was allowed to stabilize for 30 minutes before the measurement; the parameters registered for 10 seconds before CaTx application were used as control values.

The data were checked for normal distribution using the Shapiro−Wilk test. Statistical significance of the obtained results at p < 0.05 was assessed using the paired and unpaired Student’s *t* test. Data were presented as means ± SEM. Statistical data analysis was carried out using the software packages Microsoft Excel 2019 and GraphPad Prism 8.

## Results

**Figure 2.**
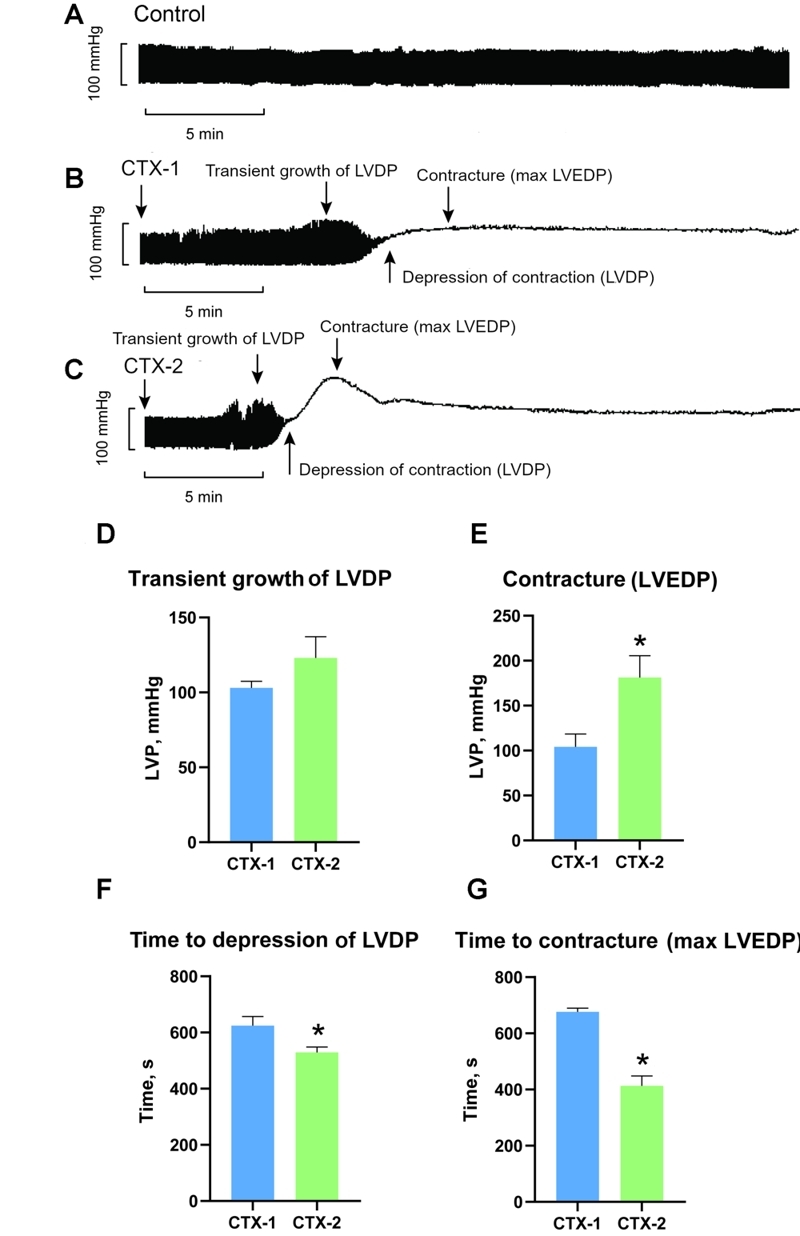
CaTX effects on the ventricular pressure parameters and effect development time. Representative traces of **(A)** untreated control and the effect of **(B)** CTX-1 and **(C)** CTX-2 at the concentration of 5 μg/mL on the contractile activity of the heart. **(D)** The magnitude of the temporary increase in LVDP and the maximum of **(E)** the developed contracture of the heart under the influence of CaTXs. The time required for complete suppression of contractile activity **(F)** in the left ventricle and **(G)** for reaching the maximum of LVEDP. CTX-1 (n = 6) and CTX-2 (n = 5). Data are presented as means ± standard error of the mean (*p < 0.05 compared to CTX-1 with unpaired two-tailed Student’s *t* test).

**Figure 3.**
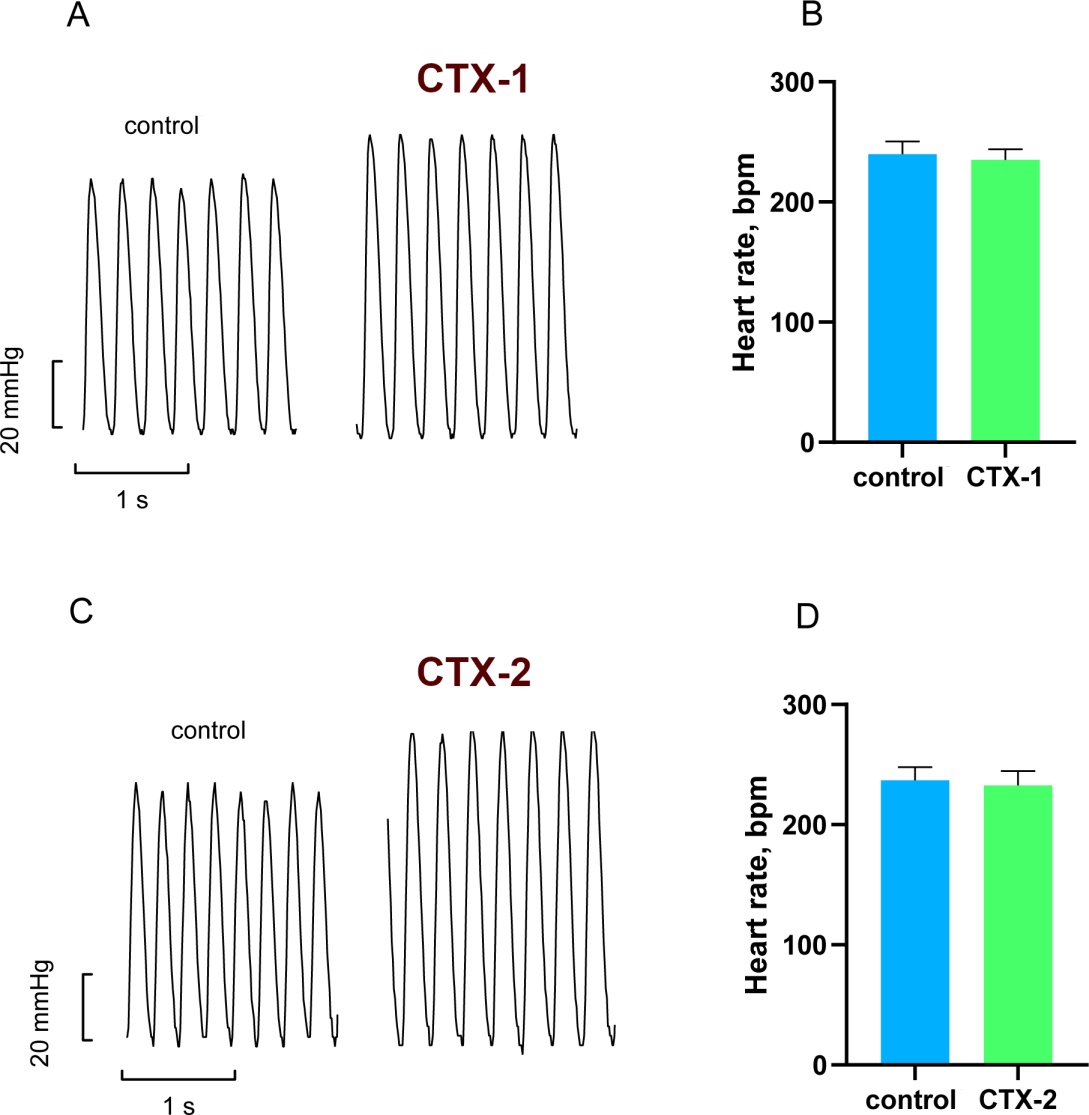
The effects of CTX-1 and CTX-2 at the concentration of 5 μg/mL on the heart rate. Representative traces of the effect of **(A)** CTX-1 and **(C)** CTX-2 on the heart rate. Histograms showing heart rate in **(B)** CTX-1 (n = 6) and **(D)** CTX-2 (n = 5) groups. BPM: beats per min. Ten-second intervals were taken at the time when the positive inotropic effect on LVDP was already at its maximum (indicated as CTX-1 and CTX-2) and just before addition of CaTX (control). Data are presented as means ± standard error of the mean. No difference was observed at *p* < 0.05 by the paired two-tailed Student’s *t* test.

To compare the action of toxins, the concentration of 5 μg/mL was chosen since at lower concentrations the effect develops fairly slowly (during 30 to 60 minutes) [9], which can complicate data interpretation due to hypoxic phenomena that may occur in the myocardium. At the same time, the use of higher concentrations can mask possible differences in the activity of toxins [15]. In the experiments with CaTXs, the hearts used were those in which the LVDP levels after the stabilization period practically did not differ and were 80 ± 2 and 82 ± 2 mm Hg for CTX-1 and CTX-2, respectively. A study of the effect of toxins on the cardiac contractile activity showed that the two toxins acted similarly (Figure 2B and 2C): after a short latency period of 6-8 minutes for CTX-1 and 3-5 minutes for CTX-2, there was a short-term increase in systolic LVDP up to 103 ± 4 mmHg under the influence of CTX-1 and up to 123 ± 14 mmHg under the influence of CTX-2. Although this increase was statistically significant compared to the LVDP value before the application of CaTXs, the effects of two CaTXs did not differ significantly. This means that both toxins increased the strength of left ventricular contractions, the effect being slightly more pronounced with CTX-2 (Figure 2D). Next, there was a decrease in LVDP up to complete cardiac arrest with a simultaneous elevation in LVEDP, which was much stronger in the case of CTX-2, being 181 ± 24 mmHg versus 104 ± 14 mmHg for CTX-1, a statistically significant difference (Figure 2E). It should also be noted that it took significantly longer for the effects to fully develop under the influence of CTX-1, compared to CTX-2. Thus, from the beginning of exposure, CTX-1 and CTX-2 suppressed cardiac contractility at 676 ± 13 and 413 ± 35 seconds and caused contracture at 624 ± 32 and 529 ± 20 seconds, respectively, differences that were statistically significant (Figure 2F and 2G). Thus, CTX-1, belonging to the S type, demonstrated lower potency in the impairment of functional heart activity than the P-type CTX-2. It should also be noted that CTX-1 and CTX-2 did not induce significant changes in heart rate at the time when the positive inotropic effect on LVDP was already at its maximum (Figure 3).

## Discussion

CaTXs are the main components of cobra venom and manifest cytotoxicity against different types of cells [16]. As discussed previously, CaTXs are classified into two types, denominated S and P, the main difference between them being in the structure of their loop 2 (Figure 1). It was shown earlier that both P- and S-type toxins interact similarly with the lipid bilayer, namely by penetrating membrane with their three loops. However, P-type CaTX inserts its hydrophobic loop II deeper into the membrane than S-type CaTX [3, 13]. Recently it was demonstrated that NS-CTX and NK-CTX, being P-type and S-type CaTXs, respectively, exhibited concentration-dependent growth inhibitory effects on human lung (A549), prostate (PC-3), and breast (MCF-7) cancer cell lines [17]. Interestingly, P-type NS-CTX was significantly more potent in inhibiting the growth of the cancer cells [17]. 

The biological effects of CaTXs include tissue necrosis, cardiac arrest and several others [18]. It should be noted that the acute toxicities of CaTXs of S- and P-types do not differ significantly, being in the range of 1-2 mg/kg for intravenous injection in mice [19, 20]. An earlier study on the effect of some cardiotoxic cobra venom fractions on the isolated rat heart provided no data on the structure of toxins comprising these fractions [5]. So far, there is no data about similarity or difference in the effects of the two types of CaTXs on the whole heart. 

To compare the effects of the S- and P-type CaTXs on the isolated rat heart, we applied two individual toxins, CTX-1 and CTX-2, purified from *Naja oxiana* cobra venom. Our experiments showed that, in general, the development of the effects of both CaTXs on the whole rat heart proceeded as described in the literature for preparations of various types of myocardial tissue - the initial increase in contraction is followed by suppression with a simultaneous increase in the resting tension [9, 21]. The available data indicate that CaTXs increase the concentrations of calcium ions in cardiomyocytes [21, 22], vascular smooth muscle cells [23], and in vascular endothelial cells [24]. The observed initial increase in the heart contractility may be explained by the rise in the intracellular concentration of Ca^2+^ ions due to increase of the extracellular Ca^2+^ influx [21]. As CTX-2 induces a stronger increase in the heart contractility than CTX-1, the intracellular rise in the concentration of Ca^2+^ ions is probably higher in the presence of this toxin. However, it should be noted that due to the positive force-frequency correlation of the rat myocardium in the range of physiological frequencies [25], an increase in the heart rate can also result in LVDP elevation. 

In our experiments, the heart rate does not increase in the presence of CaTXs (Figure 3) and, therefore, cannot account for LVDP growth. The observed subsequent diminution in LVDP and elevation in LVEDP may be a consequence of multiple processes leading to an overload of myocardial cells with Ca^2+^ and Na^+^ ions. These processes may include the activation of ion channels [26], formation of a nonselective ion channel [27], and a change in the activity of ion pumps [28, 29], ultimately leading to the irreversible cell damage and death. Recently it has been shown that CaTXs are able to penetrate plasma membrane and outer mitochondrial membrane of the cell to target anionic cardiolipin and disrupt inner mitochondrial membrane structure and bioenergetics, which may result in cardiomyocyte death [30, 31]. All these processes are dependent on the integrity of the cell or mitochondrial membrane. P-type CTX-2 disrupts the membrane more strongly than S-type CXT-1 [3] and thus may more severely influence each of these processes. 

Some differences between CTX-1 and CTX-2 in interaction with phospholipids were observed previously [30]. While both CTX-1 and CTX-2 exhibit similar biophysical effects on model membranes composed of phosphatidylcholine and cardiolipin for forming non-bilayer structures, only CTX-2 was able to form non-bilayer structures in large unilamellar membranes composed of phosphatidylcholine and phosphatidylserine [30]. Indeed, in our experiments, CTX-2 is more effective in contributing to the overload of cells with Ca^2+^ and Na^+^ ions as the increase in LVEDP is much stronger and the time to fully develop the effects is significantly shorter in the case of CTX-2. Overall, our results indicate that CTX-2 possesses a higher potency than CTX-1 in relation to damaging effects on the whole rat heart. Thus, the higher activity of CTX-2 as compared to CTX-1 demonstrated in the frog heart [8] and the papillary muscle of the rat right ventricle [9] is retained for the whole rat heart.

## Conclusion

Therefore, for the first time, we have compared the effects of two types of CaTXs on the isolated mammalian heart. Both toxins showed a similar profile of action; however, the P-type toxin CTX-2 has a higher potency. Apparently, the previously shown greater ability of this toxin to disrupt the lipid bilayer *in vitro* leads to its greater toxicity *ex vivo*.

### Abbreviations

CaTXs: cardiotoxins; CTX-1: cytotoxin (cardiotoxin) 1; CTX-2: cytotoxin (cardiotoxin) 2; LVDP: left ventricular developed pressure; LVEDP: left ventricle end-diastolic pressure; LVP: left ventricular pressure.
